# Digital Dental Models: Is Photogrammetry an Alternative to Dental Extraoral and Intraoral Scanners?

**DOI:** 10.3390/dj10020024

**Published:** 2022-02-07

**Authors:** Francesca Zotti, Luca Rosolin, Massimo Bersani, Andrea Poscolere, Davide Pappalardo, Nicoletta Zerman

**Affiliations:** 1 Department of Surgical Sciences, Paediatrics and Gynecology, University of Verona, 37134 Verona, Italy; massimo.bersani@univr.it (M.B.); nicoletta.zerman@univr.it (N.Z.); 2 Private Practice, 37134 Verona, Italy; rosolinluca.odo@gmail.com (L.R.); andreaposcolere@gmail.com (A.P.); 3 Private Practice, 35121 Padova, Italy; davidepappalardo01@gmail.com

**Keywords:** photogrammetry, digital dentistry, digital dental model, intraoral scanner, dental extraoral laboratory scanner, volumetric analysis

## Abstract

Background: 3D models are nowadays part of daily clinical practice. Photogrammetry is a brand-new method for transforming small objects into 3D models while keeping their original shape and size. The aim of this study was to evaluate the accuracy, in terms of precision and trueness, of a digital dental model acquired with photogrammetry compared with those obtained using extraoral scanners and intraoral scanners, starting from the same plaster model. Methods: A plaster model was converted into a digital model using photogrammetry, an extraoral scanner and an intraoral scanner. Different references were measured twice at a distance of 30 min for each model, on the digital models using the software Blender and on the plaster model using a calibre. The Interclass Correlation Coefficient was calculated for each pair of measurements. A volumetric analysis was performed by superimposing the digital models. The coefficient of variation was calculated. A two-way ANOVA test was conducted. Results: For each reference, the coefficient of variation was less than 3%, and the two ANOVA tests resulted in a non-significant value in both cases (*p* > 0.05). The volumetric analysis demonstrated good agreement between the models derived from the different acquisition methods. Conclusions: Photogrammetry seems to be a good method for acquiring digital models starting from a plaster model, all the methods tested seem to be good for obtaining an accurate three-dimensional digital model. Other studies are needed to evaluate clinical efficacy.

## 1. Introduction

Through the latest developments in computer science, digital technology has been widely integrated into dental practice. Patients’ photographs, radiographs, history and also dental models did not have to be physically stored but they could be stored in electronic hardware to save space in the dental office, achieving the ability to access information quickly and intuitively [1].

Many studies have been done performed to compare linear measurements of digital dental models and plaster models, concluding that these are statistically similar [2,3]. Mowever, many of these studies did not consider photogrammetry as an acquisition source for the digital dental models.

Photogrammetry has been defined by the American Society of Photogrammetry and Remote Sensing (ASPRS) as the “art, science and technology of obtaining reliable information about physical objects and the environment, through the process of recording, measuring and interpreting imagery and digital representations of energy patterns derived from non-contact sensor system” [4]. Photogrammetry could be a good method for transforming small objects into 3D models with size and shape similar to the original object [5]. In the dental and in maxillofacial fields, this source of acquisition could be useful for obtaining digital dental models from plaster models [6,7,8], to estimate facial asymmetry and to design orthognathic surgery [9], in addition to the diagnosis, case study and facial soft tissue measurements in orthodontics and dentofacial orthopaedics [10,11].

Dental extraoral laboratory scanners are used for digitalizing dental models. Such scanners include laser scanning devices that use a one-dimensional line pattern, and structured light scanning devices that use a two-dimensional light pattern [12]. Several studies have indicated that dental extraoral laboratory scanners have clinically acceptable accuracy [13,14]. Some publications have evaluated that the digital dental models acquired through the use of dental extraoral laboratory scanners have greater precision than intraoral scanners [15,16].

Dental intraoral scanners are used for acquiring a digital dental model by directly scanning teeth and intraoral tissues using specific cameras. The digital dental model is visible in real-time on a computer monitor. There are many positive aspects of digital impressions compared to traditional ones, direct digital impressions with intraoral scanners are more comfortable for the patients [17,18,19], and in some cases easier for the clinician [20,21,22,23]. Not all the issues of making traditional impressions are overcome by the use of intraoral scanners. intraoral scanners are not able to see dental tissues through periodontal tissues, so a retractor cord is needed were trying to capture subgingival margins. The presence of blood and/or saliva could also influence the success of the impressions. The assessment of the individual situation is always required to understand which technique is the best choice, for example in a completely edentulous arch, digital intraoral scanners could not take a good impression [24,25].

This study aimed to evaluate the accuracy, in terms of precision (deviation between datasets from the same acquisition method) and trueness (deviation between datasets from different acquisition methods) [15,26] of a digital dental model acquired with photogrammetry compared with those obtained using dental extraoral laboratory scanners and dental intraoral scanners starting from the same plaster model.

## 2. Materials and Methods

An alginate impression of an upper arch was taken and a plaster model was created.

### 2.1. Photogrammetry Method

**Figure 1:**
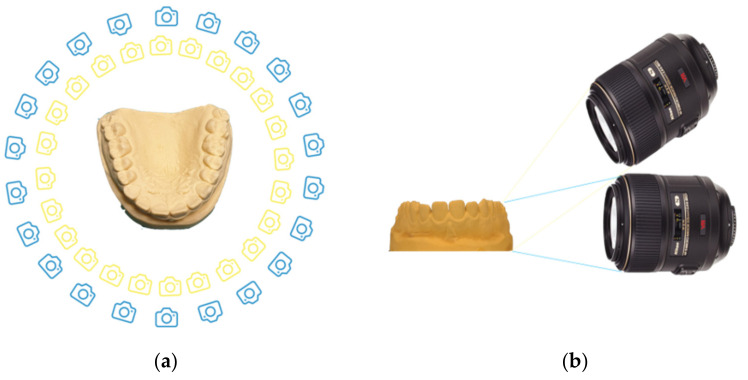
(**a**) Pattern of the realization of the photographs; (**b**) angulation of the camera: parallel to the occlusal plane and with an angle of 30°.

A total of 4 sets of 50 photos of the same plaster model were taken, turning around the model (Figure 1), consisting of:

- 25 photos parallel to the occlusal plane, and
- 25 photos with an angle of 30° from the occlusal plane.

To ensure the right angulation of 30° and 0° to the occlusal plane of the camera, a spirit level was placed on the basement, to which the camera was fixed. Twenty-five landmarks on the floor were signed by using a goniometer in order to establish the same angle for taking photographs around the model; the tripod was placed on these different landmarks in every shot. The time necessary to acquire the 50 photographs was about 25 min.

**Figure 2:**
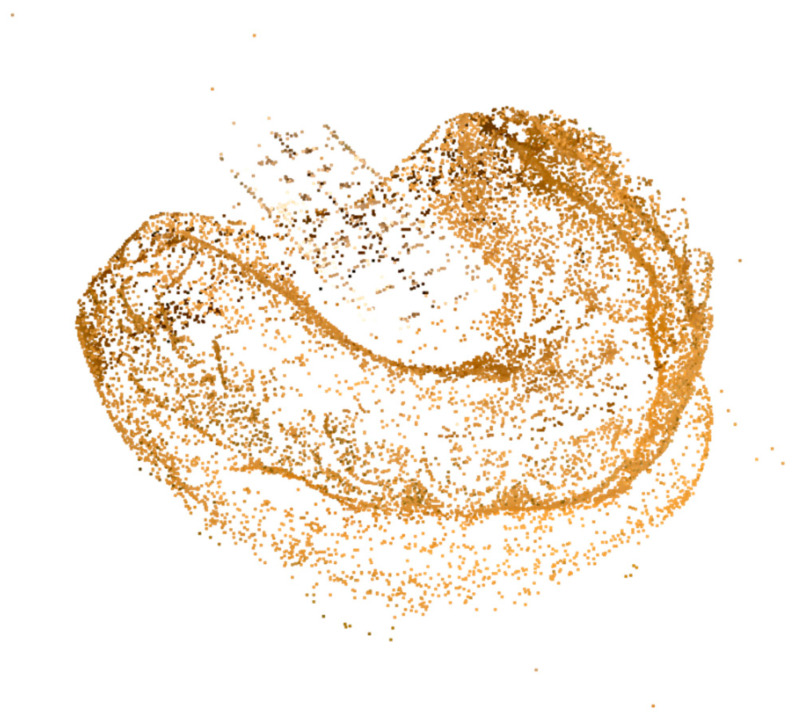
Sparse point cloud.

**Figure 3:**
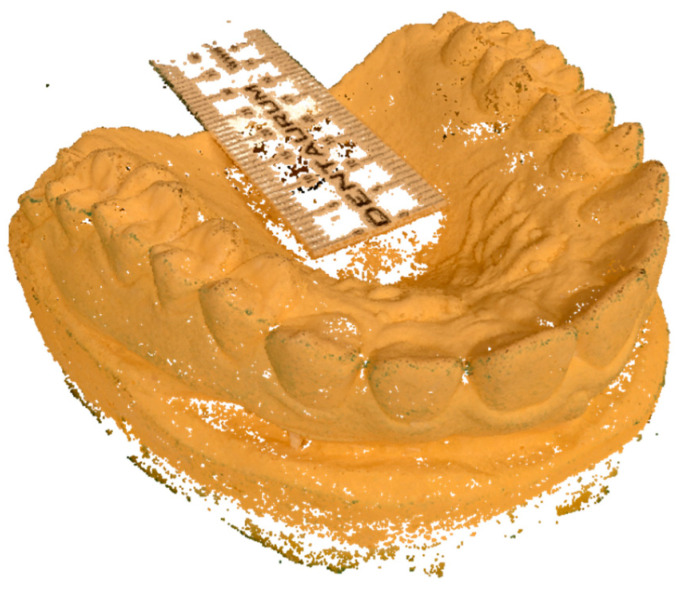
Dense point cloud.

**Figure 4:**
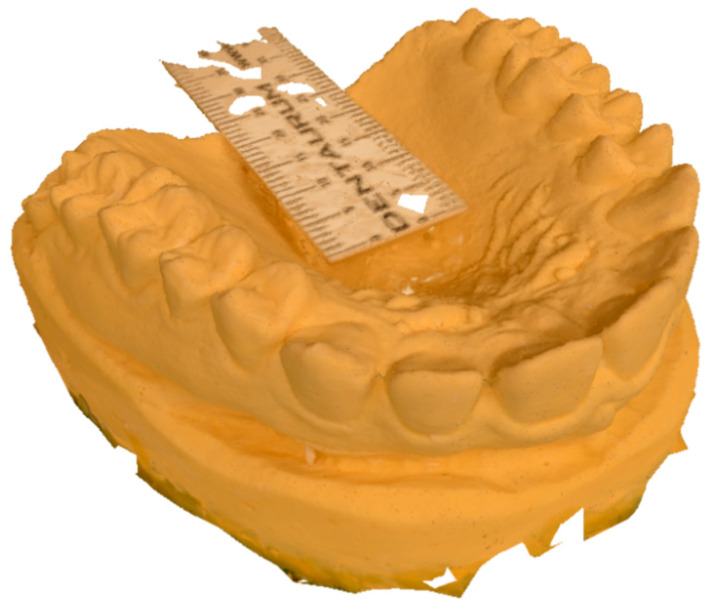
Textured mesh.

Photos were taken by a single operator using room lights. The professional camera was a Nikon D7000 (Shinjuku, Tokyo, Japan) with a Nikon AF Macro Nikkor 105 mm 1:2:8 D lens. The camera was stabilized on a tripod and set at ISO 100, f32, and an exposure time of 2.5 s. Flash and autofocus were not used. All the images were imported to 3DF Zephir Free^®^ software (3D Flow^®^, Verona, Italy) that facilitated the production of a photogrammetric reconstruction using the 50 photographs. Since a measure of reference was necessary to scale the mesh, a double decimeter was included in the photos and the photogrammetric reconstruction. The workflow performed in the software was the following:

- A new project for each set was opened, and all the photos of each set were uploaded;
- Photos were masked, using the internal software plugin “Masquerade”, to remove the background;
- A sparse point cloud was generated by the pairing of the photos. The “category” was set to “Close Range” and the pre-set was set to “Deep” (Figure 2);
- A dense point cloud was generated, setting the category to “Close Range” and the pre-set to “High Details” (Figure 3);
- A mesh was extracted from the dense point cloud by setting the category to “Close Range” and the pre-set to “High Details”;
- A textured mesh was generated by setting the category to “General” and the pre-set to “Default Single Texture”,
- The mesh was scaled using the double decimeter as a measure of reference;
- The mesh with texture was extracted from the software with the OBJ/MTL extension (Figure 4).

The OBJ file was imported into Autodesk Meshmixer (freeware, http://www.meshmixer.com, accessed on 12 December 2021) to decrease the total number of vertices and mesh faces through the “reduce” function and the “smooth” function. Then, the mesh was exported in stereolithography (STL) format.

### 2.2. Dental Extraoral Laboratory Scanner Method

The same plaster model was scanned with a dental extraoral laboratory scanner, a Zirkonzahn S600 ARTI (Zirkohnzahn GmbH, Gais, Italy). A stereolithography (STL) file was the result of the scanning process.

### 2.3. Intraoral Scanner Method

The same plaster model was scanned with an intraoral scanner, a Carestream CS3600 (Carestream, Rochester, NY, USA). A stereolithography (STL) file was the result of the scanning process.

### 2.4. Measurements

**Figure 5:**
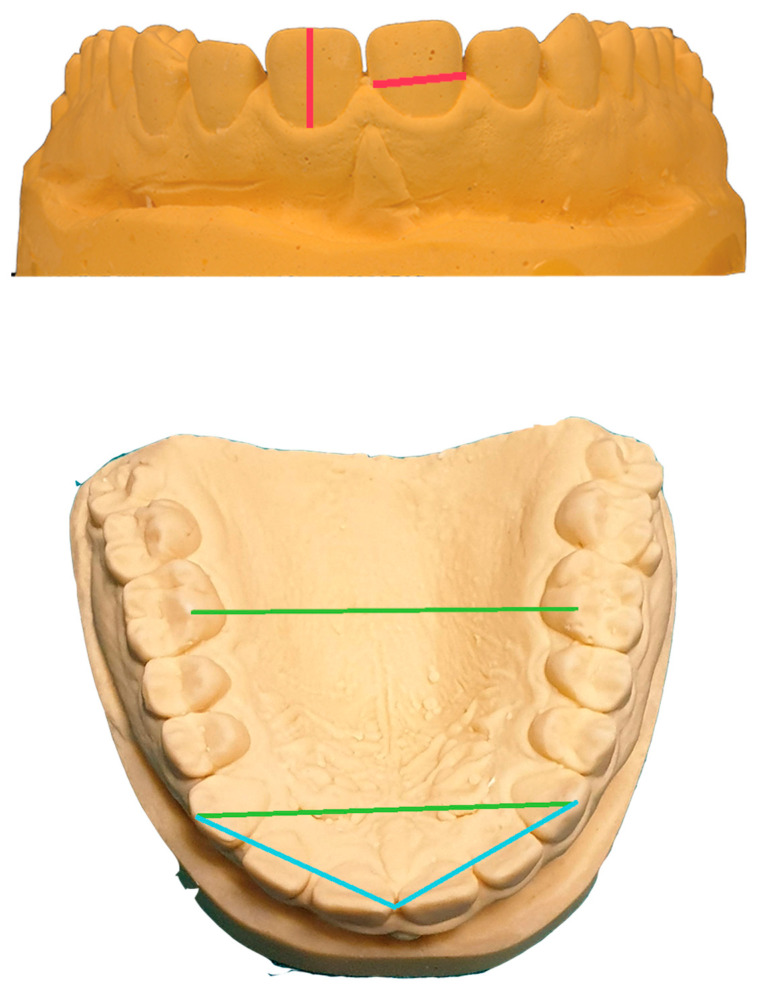
References taken for measurements.

The meshes generated through photogrammetry and the digital dental models obtained through the dental extraoral laboratory scanner and the intraoral scanner were imported into Blender Software^®^ (Blender Foundation^®^, Amsterdam, The Netherlands). Different references were measured on the plaster model (PM) and the digital models twice at a distance of 30 min from the same operator. The references were: height and width of each tooth, transverse widths between the canine cusps and the mesio-palatal cusps of the first molars, and distance between the midline and the cusps of canines (Figure 5). Mean values from all measurements were evaluated to simplify the calculation. The measurements on the plaster model were taken using a calibre and compared with those obtained from the dental digital models. The values were obtained in millimeters, with an accuracy of two decimal points.

### 2.5. Superimposition

The STL files were imported into 3D Slicer software (freeware, open-source, https://www.slicer.org). The workflow performed in the software was the following:

- A new project was created and two STL files were uploaded;
- Meshes were cut using the Easy Clip module to keep only the teeth from the second molar to the second molar;
- Meshes were approached using the “surface registration” module and setting the type of registration to “fiducial registration”;
- Meshes were superimposed using the “Surface Registration” module and setting the type of registration to “Surface Registration”;
- Mean distances and standard deviation from all the points of the meshes were measured using the “Model to Model distance” module and a VTK file with all the distances was created;
- The “Mesh Statistic” module was used to read the mean distance and the standard deviation from the saved VTK file;
- The VTK file was uploaded to the “Shape Population Viewer” module to create a color map and to view discrepancies ranging from −0.5 mm to 0.5 mm.

### 2.6. Precision of Photogrammetry

To assess the precision of photogrammetry, the deviations between datasets from the same acquisition method were evaluated. The Interclass Correlation Coefficient (ICC) was calculated for each pair of measurements made on the plaster model (PM) and each digital model created by photogrammetry (P1, P2, P3, P4). Descriptive statistics were taken, and the coefficient of variation for each reference was assessed. Small values of the coefficient of variation indicate good repeatability of the measurements within the four digital dental models created through the use of photogrammetry. The difference scores were checked for normal distribution using the Kolmogorov–Smirnov test. Differences between the PM measurements and those on the digital dental models (F1, F2, F3, F4) were evaluated by a two-way ANOVA test. All data were saved in Microsoft Excel 2010^®^ (Microsoft^®^, United States) and a database was created. Statistical tests were conducted using Medcalc software (MedCalc^®^, Mariakerke, Belgium).

### 2.7. Trueness of Photogrammetry

To assess the trueness of photogrammetry, the deviation between datasets from different acquisition methods (photogrammetry: PG; dental extraoral laboratory scanner: ES and intraoral scanner: IS) were evaluated. Descriptive statistics for each reference were calculated. The Interclass Correlation Coefficient (ICC) was calculated for each pair of measurements made on the plaster model (PM) and each digital model created by different acquisition methods (PG, ES, IS). To determine the agreement of the various acquisition methods, Bland–Altman plots were created comparing each digital dental model with the plaster model (reference model). The difference scores were checked for normal distribution using the Kolmogorov–Smirnov test. Differences between the PM measurements and those on the digital dental models (PG, ES, IS) were evaluated by a two-way ANOVA test.

The digital dental models resulting from the different acquisition methods were, furthermore, superimposed to assess differences between meshes, and a color bar was used to determine the difference between each pair of models, using a set range from −0.5 mm to 0.5 mm. The values representing the offsets for each pair of meshes were distributed normally, and a histogram chart was created to check the trend. All data were saved in Microsoft Excel 2010^®^ (Microsoft^®^, United States) and a database was created. Statistical tests were conducted using Medcalc software (MedCalc^®^, Mariakerke, Belgium), and the significance level was set at ≤0.05.

## 3. Results

### 3.1. Precision of Photogrammetry

All the measurements made on the four digital models and created using photogrammetry (P1, P2, P3, P4), and the measurements made on the plaster model were imported to Microsoft Excel 2010*^®^* and a database was created. The mean and standard deviation (SD) in mm were calculated for each reference, as was the coefficient of variation (CV). The results are reported in Table 1.

The Interclass Correlation Coefficient (ICC) was calculated for each pair of measurements made on the plaster model (PM) and each digital model created by photogrammetry (P1, P2, P3, P4). ICC was 0.99 for each pair of measurements, indicating an almost perfect agreement between the two measurements. The Two-Way ANOVA test was performed on all the values obtained from the measurements on the plaster model and the 4 digital models obtained through the use of photogrammetry. The test results were not statistically significant (*p*-value = 0.98), showing no differences between the measurements carried out on plaster models and those on digital dental models.

### 3.2. Trueness of Photogrammetry

**Figure 6:**
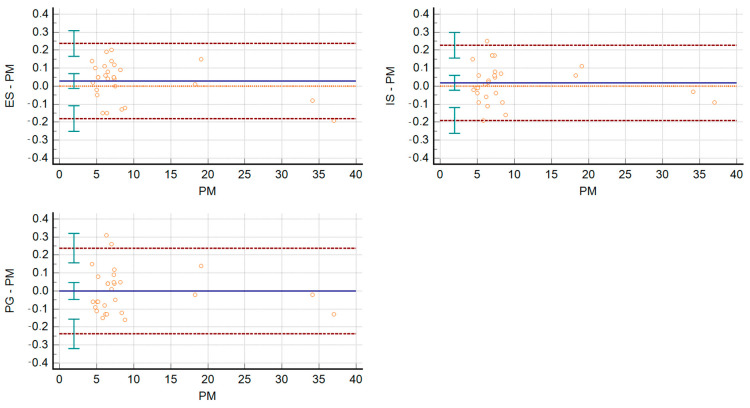
Bland–Altman graphs to visualize the agreement of the measurements taken on the 3 digital models with those taken on the plaster model.

All the measurements made on the three digital models created using three different acquisition methods (PG, ES, IS), and the measurements made on the plaster model were imported to Microsoft Excel 2010*^®^,* and a database was created. The mean and SD in mm were calculated for each reference. The ICC was also calculated for each pair of measurements made on the plaster model (PM) and on each digital model created using the 3 different acquisition methods (PG, ES, IS). ICC was 0.99 for each pair of measurements. The Bland–Altman graphs created to determine the agreement between the measurements made on the three digital dental models compared to the measurements made on the plaster model are shown below (Figure 6).

The two-way ANOVA test was performed on all the values obtained from the measurements of the plaster model and of the three digital models obtained using PG, IS and ES. The test was not statistically significant, with a *p*-value = 0.20, showing no differences between the four methods of acquisition in terms of reproducibility. The three digital dental models created using the three different acquisition methods were superimposed according to a Surface Registration algorithm. For each pair of models, all the distances present between the two models were exported. From the volumetric analysis, more than 100,000 distances were exported (representing the difference between the two meshes). For each pair of digital dental models, these distances were distributed in a normal way and histograms were created for each pair of meshes. For the various superimpositions, the mean ± SD (mm) of the differences between the two meshes were:

- Photogrammetry/Dental extraoral laboratory scanner: −0.02 ± 0.077;
- Photogrammetry/Intraoral Scanner: 0.009 ± 0.087;
- Intraoral Scanner/Dental extraoral laboratory scanner: 0.031 ± 0.058.

**Figure 7:**
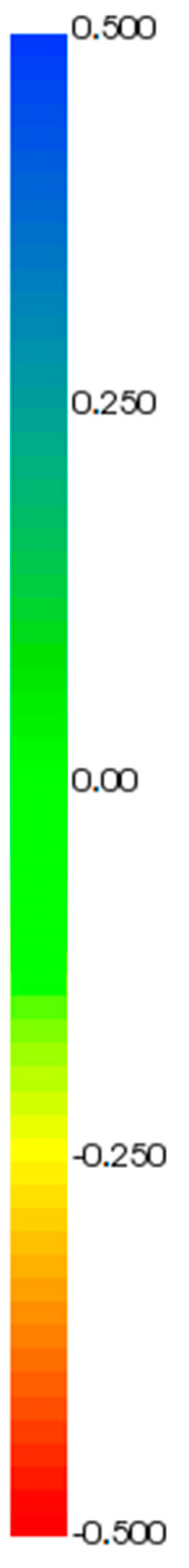
Color bar used to visualize discrepancies between digital dental models.

**Figure 8:**
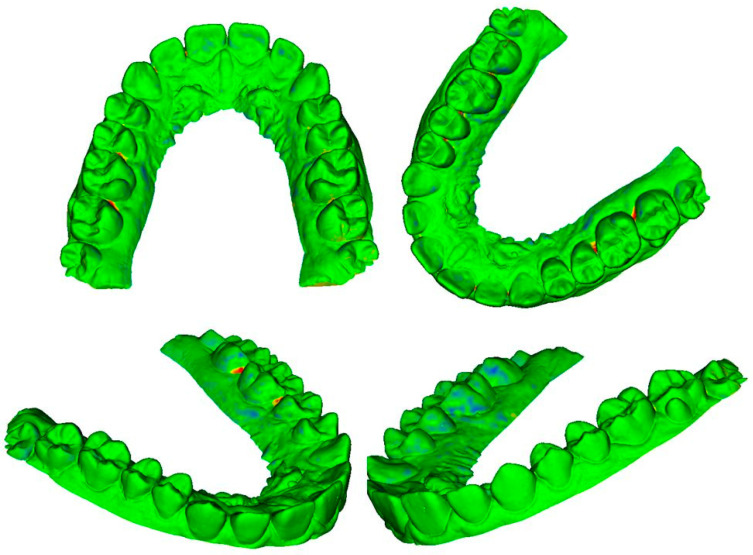
Superimposition between photogrammetry and dental extraoral laboratory scanner.

**Figure 9:**
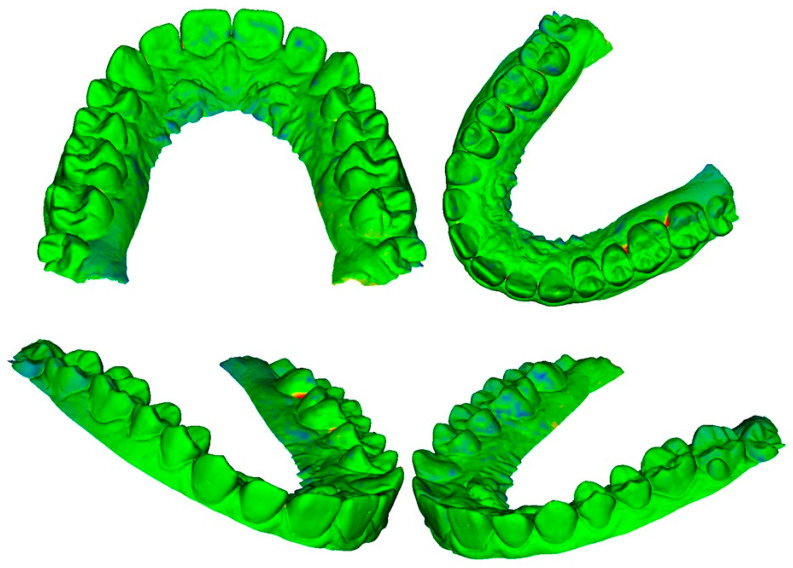
Superimposition between photogrammetry and intraoral scanner.

**Figure 10:**
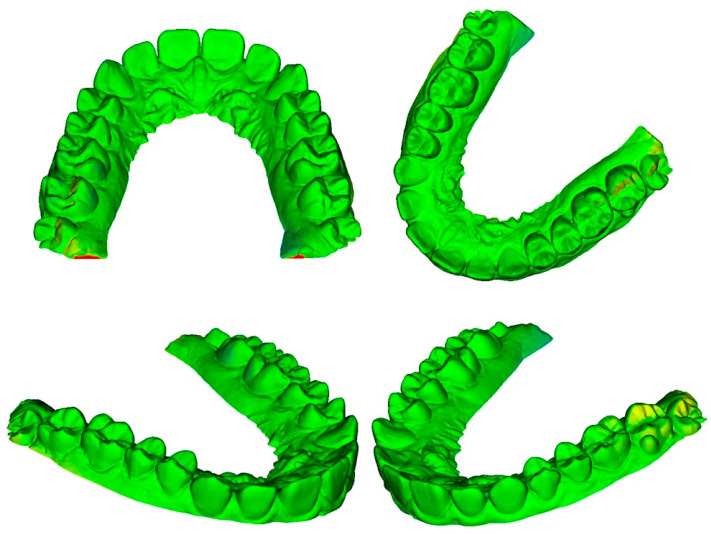
Superimposition between dental extraoral laboratory scanner and intraoral scanner.

**Figure 11:**
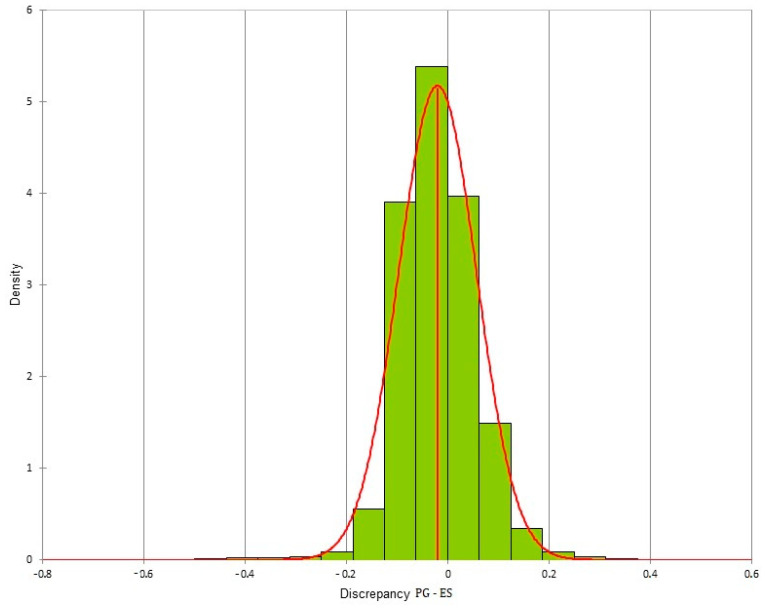
Histogram of the discrepancy between photogrammetry and dental extraoral laboratory scanner.

**Figure 12:**
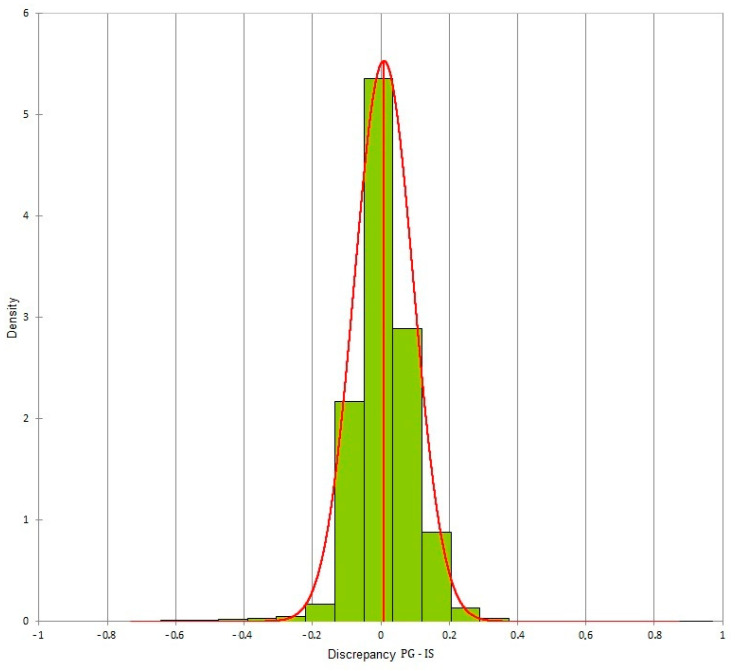
Histogram of the discrepancy between photogrammetry and intraoral scanner.

**Figure 13:**
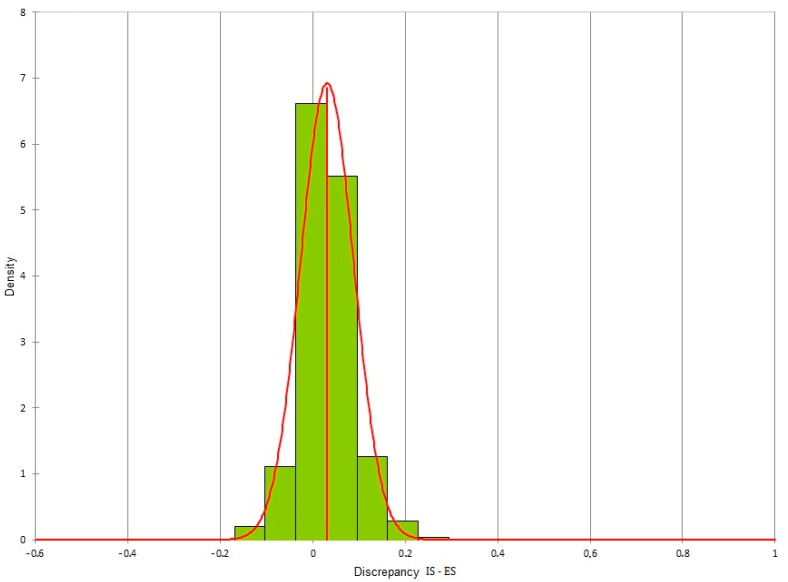
Histogram of the discrepancy between dental extraoral laboratory scanner and intraoral scanner.

The differences for each pair of digital dental models could be viewed through colored meshes where the greatest differences were visible. The range used varied from −0.5 mm to 0.5 mm. The color bar used is reported in Figure 7, the superimposed meshes are reported in Figure 8, Figure 9 and Figure 10, and the histograms are reported in Figure 11, Figure 12 and Figure 13.

## 4. Discussion

One of the reasons this study came about was the need to validate a method of digitalizing a plaster model that can also be used where there is no possibility to access more sophisticated technological equipment. Different software packages were used for the realization of a digital model using photogrammetry. However, the priority was given to open source software, for the creation of the digital dental model, for its treatment of linear measurements, and for the ability to superpose the models to determine their accuracy. This choice was dictated by the need to assess the feasibility of the model to obtain results at a low financial cost.

For many years, the use of photogrammetry was difficult because of the strict need to use special equipment to take images and processing them [6,27]. As some studies reported [28,29], in recent years, due to the advancement of technology in the field of digital photography and the creation of specific software, photogrammetry has become an accurate, reliable, and economically accessible method for most dentists, and it is now becoming an interesting resource in the field. The accuracy of photogrammetry was evaluated in terms of precision (deviation between datasets from the same acquisition method) and trueness (deviation between datasets from different acquisition methods) [15,26]. Regarding the precision of the photogrammetry, the results of the present study were found to agree with those obtained by Stuani et al. [6]. Moreover, the measurements made on the digital models derived from photogrammetry were consistent with the measurements made on the plaster model taken as the reference. The Interclass Correlation Coefficient evaluated between each pair of measurements taken on each digital dental model obtained by photogrammetry and on the plaster model was equal to 0.99, demonstrating an almost-perfect concordance between the two measurements and therefore no errors in the measurement of the models. The coefficient of variation calculated for each reference between the values deriving from the measurements on the plaster model and the digital dental models P1, P2, P3, and P4 was always less than 3%, indicating the good repeatability of this method. The same result was demonstrated by Xiaoming Fu et al. [30] who applied the photogrammetric technique using a total of 72 photographs taken around the model. The ICCs calculated for linear measurements on digital dental models were found to be between 0.879 and 0.998, also indicating almost-perfect agreement in this case. The percentage coefficient of variation ranged from 0.165% to 6.731%, indicating the good repeatability of this method. Xiaoming Fu et al. also concluded that the measurements made on digital dental models are to be considered reproducible [30].

Evaluating the trueness of the photogrammetry when compared with intraoral scanning and extraoral scanning, no statistically significant differences were noticed between the values resulting from the linear measurements on the plaster model and the digital dental models from the 3 different acquisition methods tested. The non-significance of this test can indicate how the linear dimensions obtained on a digital dental model are not different even if they are obtained from different acquisition methods. It also indicates how the overall dimensions regarding width and height of the crown, as well as the transversal measurements between canines and between molars, can be evaluated using software that handles reconstructions derived from photogrammetric acquisitions.

Some previous studies showed that photogrammetry can reproduce the shape of the digitized object and obtain measurements comparable to those performed on models obtained from high-resolution 3D scans [9,31,32]. Despite the ability of photogrammetry to recreate a digital dental model with an excellent shape and size of the dental elements, the small-scale representation of the occlusal topography was poor compared to the three-dimensional models obtained with the other acquisition methods and compared to the plaster model. The use of only one digital dental model for each acquisition method was one of the limitations of this study. By generating a series of digital dental models for each acquisition method, the random variability would be increased and the statistical tests used could be more accurate. Surely, in these terms, a high-performance impression material could be evaluated to obtain more accurate dental plaster models. Additinally, different dental plasters could be tested to assess the weight of different variables involved in plaster model creation.

A volumetric study was performed on each pair of digital dental models and it was based on the analysis of the gaps present between the two meshes. The averages deriving from more than 100,000 values obtained as a point-by-point deviation from each pair of meshes denoted that they differed from each other, on average, by a maximum value of 31 μm. In the photogrammetric mesh, the vestibular area was characterized by greater precision of details than the palatal area. This might have been caused by the limited number of photographs of the palatal sector. Most of the photographs taken parallel to the occlusal plane portrayed only the vestibular part of the plaster model, while the palatal side, especially the mesio-palatal sector of the dental elements, was captured in a smaller number of images and only in those taken with the inclination of 30° from the occlusal plane. In these areas, greater gaps were noticed when the photogrammetric mesh was superimposed on the other two meshes. This lower precision in the palatal sector could be overcome by taking a greater number of photographs of the plaster model and by paying more attention to its palatal side.

Regarding the shape and size of the occlusal side, not many deviations from the volumetric analysis were highlighted, demonstrating a good ability of photogrammetry in obtaining and recreating the sizes of the cusps and incisal areas, both in terms of shape and form [7].

With all this in mind, we can speculate on the value of the photogrammetry for obtaining accurate digital reproductions of a plaster model; this method is of great importance considering the inherent possibility of sharing files and to work on them to plan treatment solutions. We are well aware that process of taking all the 50 photographs required for the digitalization of the cast represents a significant cost of time. However, this tool allows dentists to avoid the economic expense of a digital scanner and reduces the necessity to obtain a extraoral scanner and therefore to have an external lab. This issue plays an important role when economic sources are limited. Of course, marketing offers high-performant possibilities in this regard, however, this specific technique could be of a great importance where used by undergraduate students and young dentists, who might not be able to immediately acquire expensive technologies.

With this in mind, given the accurate results obtained by photogrammetry technique, we might assume that the advantages of this technology lie in its low cost. Furthermore, considering that this tool could be easily managed by any young, untrained students able to take photographs, photogrammetry presents a viable method to acquire digital dental models without using expensive and difficult to obtain technologies. We are well aware that digital scanners are, nowadays, available in most of the dental offices. Nevertheless, not all universities or undergraduate programs have one. Using this method, young dental students could deal with digital dental workflows during their training, even if they do not have access to all the technologies available in the market. Therefore, this method has significant potential value for young practitioners. 

The viability of introducing photogrammetry to create digital dental models could lead to the future possibility of digitally manage these models in planning digital previews of aesthetic treatments or to set up orthodontic therapies and simulate prosthetic procedures using open-source software and with an easy-to-use workflow. Further perspectives are represented, in our opinion, by the opportunity to use these plans to 3D print mock-up solutions or provisional dental elements, even if we are well aware that further evaluations and tests of techniques and materials are required to achieve these goals.

## 5. Conclusions

Photogrammetry seems to be a good and aceessible method for acquiring digital dental models, with excellent quality achieved when starting from a plaster model.

With these findings in mind, we might assume that photogrammetry is a viable means to acquire digital models at minimal expense. This leads to the possibility of using photogrammetry based on open-source software, as doing so would be within the reach of students and young dentists not yet able to acquire expensive digital tools.

The feasibility of using this technology to become familiar with the digital workflows in dentistry represents a great opportunity to develop skills that are becoming increasingly more important in clinical practice. 

## Figures and Tables

**Table 1:** Mean and SD in mm and Percentage Coefficient of Variation of measurements taken on 4 digital models created using Photogrammetry.

Reference	Mean	SD	CV (%)
Height 1.6.	4.44	0.01	0.28
Height 1.5.	4.66	0.08	1.65
Height 1.4.	6.02	0.02	0.42
Height 1.3.	7.44	0.02	0.30
Height 1.2.	5.95	0.10	1.71
Height 1.1.	7.41	0.07	0.99
Height 2.1.	7.54	0.03	0.35
Height 2.2.	6.47	0.11	1.76
Height 2.3.	8.23	0.10	1.22
Height 2.4.	6.44	0.14	2.13
Height 2.5.	5.01	0.07	1.30
Height 2.6.	4.57	0.02	0.37
Width 1.6.	8.60	0.07	0.80
Width 1.5.	5.63	0.09	1.60
Width 1.4.	5.11	0.02	0.49
Width 1.3.	7.04	0.04	0.57
Width 1.2.	6.47	0.13	2.04
Width 1.1.	7.36	0.04	0.48
Width 2.1.	7.37	0.08	1.02
Width 2.2.	6.37	0.13	2.00
Width 2.3.	7.29	0.06	0.89
Width 2.4.	5.29	0.04	0.75
Width 2.5.	4.97	0.06	1.23
Width 2.6.	8.28	0.02	0.23
Transverse 1.3.–2.3.	34.11	0.13	0.37
Transverse 1.6.–2.6.	36.78	0.17	0.47
Midline—1.3.	18.24	0.14	0.77
Midline—2.3.	19.17	0.16	0.82

## Data Availability

Not applicable.
